# An Intervention for the Transition From Pediatric or Adolescent to Adult-Oriented HIV Care: Protocol for the Development and Pilot Implementation of iTransition

**DOI:** 10.2196/24565

**Published:** 2021-04-07

**Authors:** Amanda E Tanner, Nadia Dowshen, Morgan M Philbin, Kelly L Rulison, Andres Camacho-Gonzalez, Susan Lee, Shamia J Moore, J Dennis Fortenberry, Sophia A Hussen

**Affiliations:** 1 Department of Public Health Education University of North Carolina Greensboro Greensboro, NC United States; 2 Craig-Dalsimer Division of Adolescent Medicine Children’s Hospital of Philadelphia Philadelphia, PA United States; 3 Department of Pediatrics Perelman School of Medicine University of Pennsylvania Philadelphia, PA United States; 4 Department of Sociomedical Sciences Columbia University Mailman School of Public Health New York, NY United States; 5 Department of Human Development and Family Studies The Pennsylvania State University State College, PA United States; 6 Department of Pediatrics Division of Infectious Diseases Emory University School of Medicine Atlanta, GA United States; 7 Hubert Department of Global Health Emory University Rollins School of Public Health Atlanta, GA United States; 8 Division of Adolescent Medicine Indiana University School of Medicine Indianapolis, IN United States; 9 Department of Medicine Division of Infectious Diseases Emory University School of Medicine Atlanta, GA United States

**Keywords:** HIV, mHealth, transition to adult care, young adult, feasibility studies, retention in care, control groups, United States, telemedicine, HIV infections, mobile phone

## Abstract

**Background:**

In the United States, adolescents and young adults are disproportionately affected by HIV and have poorer HIV-related health outcomes than adults. Health care transition (HCT) from pediatric or adolescent to adult-oriented HIV care is associated with disruptions to youths’ care retention, medication adherence, and viral suppression. However, no evidence-based interventions exist to improve HCT outcomes for youth living with HIV.

**Objective:**

There are 2 phases of this project. Phase 1 involves the iterative development and usability testing of a Social Cognitive Theory–based mobile health (mHealth) HIV HCT intervention (*iTransition*). In phase 2, we will conduct a pilot implementation trial to assess *iTransition*’s feasibility and acceptability and to establish preliminary efficacy among youth and provider participants.

**Methods:**

The iterative phase 1 development process will involve in-person and virtual meetings and a design team comprising youth living with HIV and health care providers. The design team will both inform the content and provide feedback on the look, feel, and process of the *iTransition* intervention. In phase 2, we will recruit 100 transition-eligible youth across two clinical sites in Atlanta, Georgia, and Philadelphia, Pennsylvania, to participate in the historical control group (n=50; data collection only) or the intervention group (n=50) in a pilot implementation trial. We will also recruit 28 provider participants across the pediatric or adolescent and adult clinics at the two sites. Data collection will include electronic medical chart abstraction for clinical outcomes as well as surveys and interviews related to demographic and behavioral characteristics; Social Cognitive Theory constructs; and intervention feasibility, acceptability, and use. Analyses will compare historical control and intervention groups in terms of HCT outcomes, including adult care linkage (primary), care retention, and viral suppression (secondary). Interview data will be analyzed using content analysis to understand the experience with use and acceptability.

**Results:**

Phase 1 (development) of *iTransition* research activities began in November 2019 and is ongoing. The data collection for the phase 2 pilot implementation trial is expected to be completed in January 2023. Final results are anticipated in summer 2023.

**Conclusions:**

The development and pilot implementation trial of the *iTransition* intervention will fill an important gap in understanding the role of mHealth interventions to support HCT outcomes for youth living with HIV.

**International Registered Report Identifier (IRRID):**

DERR1-10.2196/24565

## Introduction

Adolescents and young adults are disproportionately affected by HIV in the United States compared with their adult counterparts [1]. Youth living with HIV aged 18 to 24 years face significant barriers to continuous care engagement. Recent research suggests that only 43% of youth living with HIV have received any HIV medical care and that only 33% are virally suppressed [1]. These challenges are accentuated during the health care transition (HCT) from pediatric or adolescent to adult-oriented care [2,3]. Every youth living with HIV in pediatric or adolescent care must transition to adult care, and 25,000 youth living with HIV in the United States are projected to undergo HCT in the decade leading up to 2025 [4]. In the United States, HIV-related HCT typically occurs around the age of 24 years [5] and is often associated with disruptions to care retention, medication adherence, and viral suppression [6]. In the 1 to 2 years following expected HCT, less than 40% of youth are linked to adult care and only 50% to 60% of those linked remain engaged in care [6-9]. A seamless HCT experience can facilitate continuous care retention and viral suppression in youth living with HIV, with implications for morbidity, mortality, and public health [10]. Although multilevel barriers to HCT exist [11], existing research focuses primarily on single-site protocols that have not been rigorously evaluated, and as such, no evidence-based interventions exist to improve HCT outcomes for youth living with HIV [4,5].

Mobile health (mHealth) interventions are used to improve medication adherence, care retention, and viral suppression among youth living with HIV [12-15]. mHealth interventions are well suited for youth living with HIV: youth use the internet more than any other age group, and over 90% own smartphones [16]. In addition, providers frequently use mobile platforms in health care settings to facilitate provider-provider and patient-provider communication and to standardize clinical practices [17-19]. Therefore, an mHealth platform is an ideal vehicle for a novel multilevel intervention to improve HCT, with a high likelihood of adoption by youth living with HIV *and* their pediatric or adolescent and adult providers.

Theory-informed mHealth interventions can improve health outcomes for youth living with HIV, and Social Cognitive Theory [20] is one of the most widely used theoretical frameworks in mHealth behavioral interventions [21,22]. Social Cognitive Theory helps to articulate dynamic interactions that occur during HCT—between environment (clinical environments), behavior (care engagement through HCT), and personal factors (including self-efficacy for facilitating HCT and knowledge of the HCT process)—and is helpful in understanding the multilevel challenges associated with HCT. Accordingly, this paper describes the development and evaluation protocol for *iTransition*, a Social Cognitive Theory–based mHealth intervention to improve HCT processes for youth living with HIV and their pediatric or adolescent and adult care providers.

## Methods

### Ethics Statement

The Institutional Review Board (IRB) at the Children’s Hospital of Philadelphia (CHOP) is the IRB of record for all the participating institutions (CHOP, Emory University, and the University of Carolina Greensboro). The CHOP IRB has reviewed and approved all the procedures outlined in this protocol. The study is registered with ClinicalTrials.gov (NCT04383223).

### Study Settings

This protocol is being implemented at high-volume HIV care centers that frequently transition youth living with HIV from pediatric or adolescent to adult-oriented care. The Grady Infectious Disease Program in Atlanta, Georgia, is affiliated with a large public safety net health system and is among the largest HIV care centers in the United States, serving approximately 6000 patients per year, 10% of whom are youth living with HIV under 25 years of age. It contains pediatric or adolescent and adult-oriented clinic spaces in the same building but has no formal HCT protocol. CHOP, located in Philadelphia, Pennsylvania, contains 2 academic hospital-based HIV clinics that transition youth living with HIV to multiple adult clinics, including infectious disease clinics at the Hospital of the University of Pennsylvania and the Penn Presbyterian Medical Center. CHOP has a formal HCT protocol that includes guided pre-HCT visits to adult clinics with a pediatric or adolescent social worker. Piloting *iTransition* in clinics in Atlanta and Philadelphia with different HCT models (eg, one colocated vs multiple adult clinic options) enhances the likelihood of generalizability to other HIV clinical care settings.

### Intervention Overview

*iTransition* is grounded in Social Cognitive Theory and builds on our team’s extensive HCT work [6,7,11,23,24]. Broadly, Social Cognitive Theory outlines the dynamic interplay, or *reciprocal determinism*, between environmental, behavioral, and personal factors. Specifically, Social Cognitive Theory posits that these factors interact through constructs, including self-management skills, outcome expectancy, self-efficacy, cues to action, and behaviors, each of which forms intervention targets and situates our approach to improving HCT for youth living with HIV. Our previous research outlined 4 domains of successful HCT [6] that align with these constructs and will be addressed in *iTransition* (Table 1): (1) preparation of youth living with HIV for autonomous disease management, (2) effective interclinic provider communication, (3) enhancement of youth living with HIV’s connectedness to adult clinics, and (4) implementation of formalized HCT protocols in pediatric or adolescent and adult clinics. Intervention content and strategies will include educational modules, interactive activities (eg, quizzes), relationship development strategies (eg, youth and provider profiles), and communication (eg, individual and group chat features).

In addition, we draw on supportive accountability theory [25], which posits that human interactions with coaches—here, providers serving as Transition Champions—provide an essential complement to mHealth interventions that enhance uptake for users. In phase 2, Transition Champions will assist other participants with using the *iTransition* app, encourage utilization by their peers (providers) and patients (youth), and promote integration of *iTransition* use into clinical workflow.

**Table 1:** Domains of health care transition success, Social Cognitive Theory constructs, and intervention strategies.

Domains of HCT^a^ success	Measurable social cognitive theory constructs	*iTransition* intervention strategies (target audience)
Preparation of YLH^b^ for autonomous disease management	Self-management skills Self-efficacy	Youth-friendly educational modules and readiness assessments (youth) HCT educations modules (providers) Interactive case scenarios (youth and providers)
Enhancing YLH’s connectedness to adult clinics	Self-management skills Self-efficacy	Motivational messages (youth) TC^c^ interactions (providers) Online support forum (youth)
Effective between clinic provider communication	Self-efficacy Behavior	Reminders and updates about patients going through HCT process (providers) Communication between adult and pediatric or adolescent providers (providers)
Implementation of formalized HCT protocols in pediatric or adolescent and adult clinics	Behavior Cues to action	Clinic-level implementation (youth) TC intervention (providers)

^a^HCT: health care transition.

^b^YLH: youth living with HIV.

^c^TC: Transition Champion.

### Study Design

Phase 1 of this study, *iTransition* intervention development, uses a participatory, theoretically driven approach. In phase 2, we will conduct a pilot implementation trial of *iTransition* in Atlanta and Philadelphia, comparing post-HCT clinical HIV outcomes—adult care linkage (primary), care retention, and viral suppression (secondary)—between youth participants in our intervention with historical control groups. See Multimedia Appendix 1 for *iTransition* screenshots.

#### Phase 1: Intervention Development

**Figure 1:**
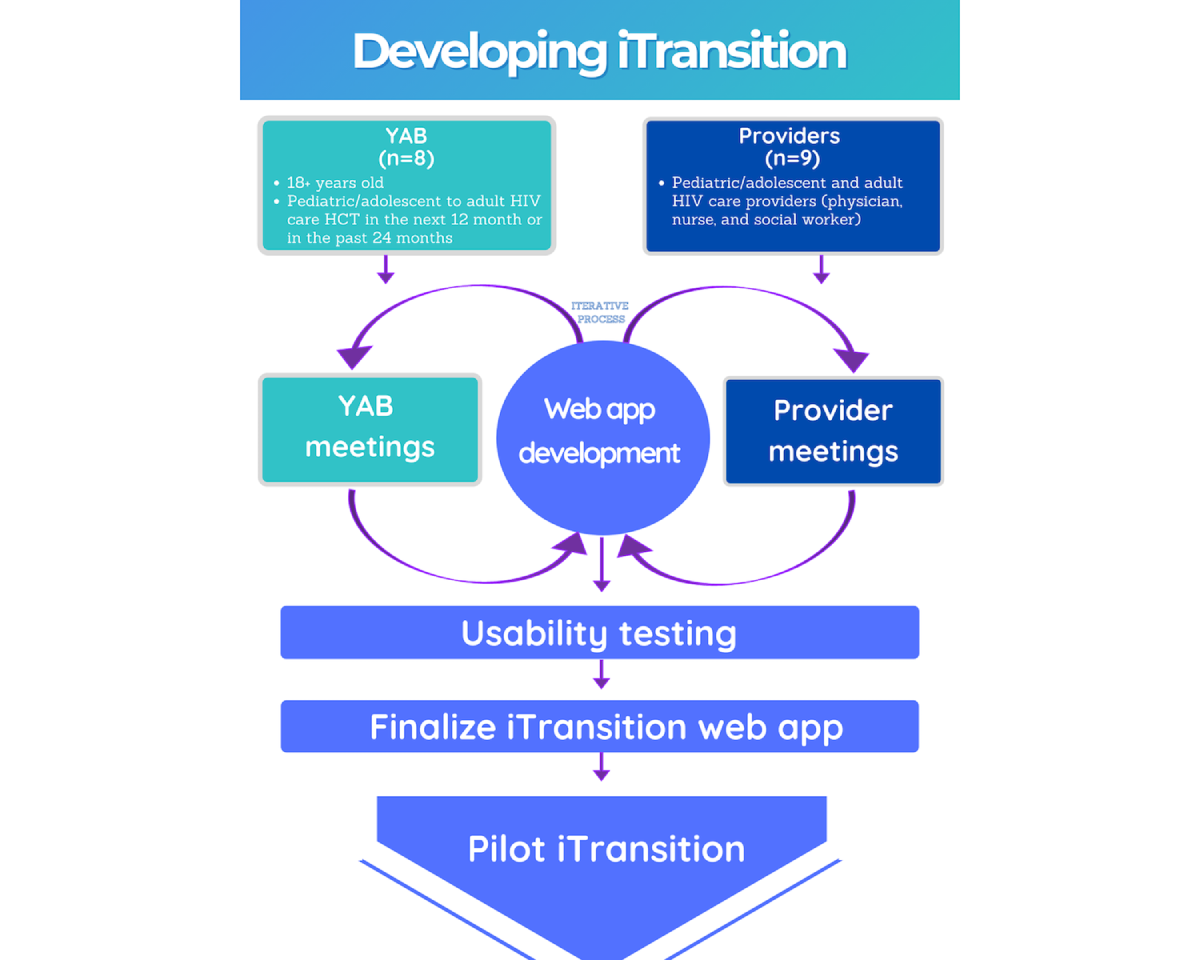
iTransition development. HCT: health care transition.

To develop *iTransition,* we will work collaboratively and iteratively with a design team that includes pre- and post-HCT youth (n=9) and pediatric or adolescent and adult-oriented HIV care providers (n=8) in Atlanta and Philadelphia (Figure 1). The provider participants in the design team will also serve as Transition Champions during the phase 2 implementation trial. On the basis of the input from the design team, we will work with our technology partner, Pattern Health, to create intervention content in the youth-facing mobile application (ie, *app*) and the provider-facing console. We will present ideas (including app and console demonstrations) to the design team members to solicit feedback for revisions on content, format, and presentation until the app is completed and ready for usability testing. This process will include regular in-person and virtual design team meetings. Once the completed mHealth intervention is built, we will conduct usability testing with at least five youth living with HIV and five providers to get further feedback on the functionality and app use experience. Specifically, these youth and providers will be asked to use *iTransition* and respond to a series of assessment questions aligned with several of the user experience honeycomb constructs—usable, useful, valuable, desirable, and credible [26,27].

#### Phase 2: Pilot Implementation Trials

**Figure 2:**
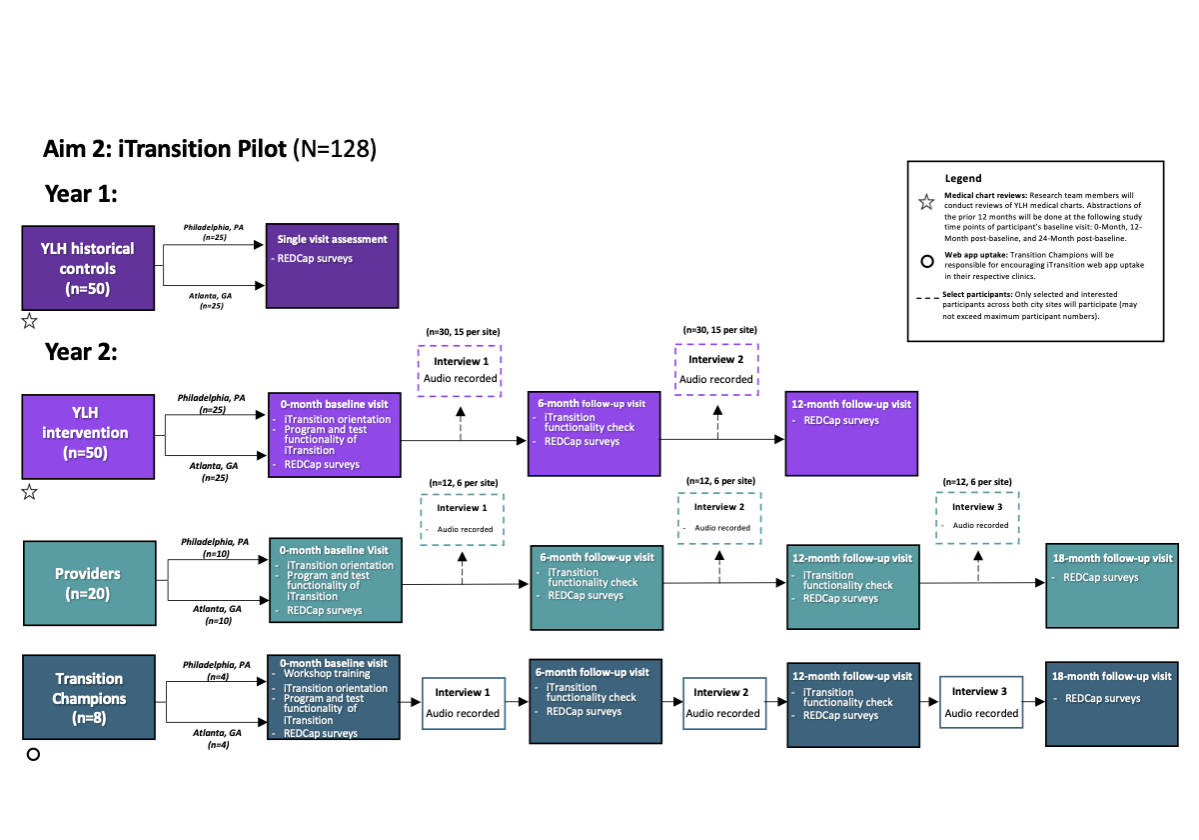
Phase 2 activities by participant type.

In phase 2, we will conduct a pilot implementation trial of *iTransition* with 100 transition-eligible (ie, within 6 months of anticipated HCT) youth living with HIV recruited by staff in participating pediatric or adolescent HIV clinics and 28 providers (inclusive of the 8 Transition Champions recruited in phase 1) from pediatric or adolescent and adult HIV clinics (Figure 2). Due to the challenges of randomizing involvement in a clinic-wide intervention, we will compare our intervention group with a historical control group that will be completely recruited and enrolled before implementation of the *iTransition* intervention. We will recruit the historical control group (n=50) from both study sites (approximately 25 per site). Following the recruitment of our historical control group, we will recruit intervention youth (n=50; approximately 25 per site) and the remaining 20 providers. We will compare the youth historical control and intervention groups in terms of their HCT outcomes. Our primary outcome is linkage to adult care, defined as attending one HIV care visit at the adult clinic. Our secondary outcomes are care engagement at the adult clinic (ie, at least two visits within 12 months, at least 90 days apart) and viral suppression (defined as less than 200 copies/mL) 1 year after baseline. We will also assess the use, acceptability, and feasibility of *iTransition* among youth and the provider and Transition Champion participants.

### Participants

For phase 1, we will recruit approximately 10 youth and 8 providers for our design team. Phase 2 will include a total of 128 participants: 100 transition-eligible youth (50 for the intervention group and 50 for the historical control group), 20 providers (approximately 10 from each city site) who work with transitioning youth living with HIV, and the 8 design team providers (recruited in phase 1) who will function as Transition Champions provider participants. Historical control youth will participate for 1 day, intervention group youth will participate for 12 months, and all providers (including Transition Champions) will participate for 18 months.

#### Youth Historical Control Group

Inclusion criteria include (1) living with HIV, (2) age 18+ years, (3) expected to undergo HCT within the next 6 months, and (4) enrolled in care at participating clinics in Atlanta and Philadelphia. Potentially eligible youth will be identified through chart review in collaboration with clinic staff and approached by a research assistant during or after regular HIV care visits at the pediatric or adolescent clinic (face-to-face or during telehealth visits). Those who express interest will be consented (including signing a consent form for the release of medical information) and screened to determine eligibility. Those who enroll will complete a single cross-sectional survey before HCT, and they will receive US $25 for their time.

#### Youth Intervention Group

Following the recruitment and participation of the historical control group, we will recruit youth living with HIV for the intervention group. Inclusion criteria for the intervention group will be identical to the historical control group with the additional criteria that they must own a smartphone or tablet and report consistent internet access (defined as no lapse >24 hours in the last 6 months). Youth without a device are not eligible for this pilot trial; larger future trials will provide access to devices as needed. Similar to the historical control group, a research assistant will invite potentially eligible youth to participate during or after regular HIV care visits at the pediatric or adolescent clinic (face-to-face or during telehealth visits). Those who express interest will provide consent (including the release of medical information) and be screened to determine eligibility. Enrolled participants will complete the same baseline survey as the historical control group at the first study visit, during which time they will also be oriented to the *iTransition* app and create a user account. Over the 12-month intervention period, youth intervention participants will complete additional surveys at 6 and 12 months, and a subset (up to 30 youth) will be invited to participate in qualitative interviews based on *iTransition* use (high and low/no). Overall, participants could receive US $75 to US $125 (US $25 per study visit or interview) for their time if they complete all assessments (baseline, 6-month, and 12-month assessments and up to 2 qualitative interviews).

#### Provider Intervention Group

Inclusion criteria for the provider intervention group include (1) staff members at participating clinics, (2) work with transitioning youth, and (3) access to the internet via any device (eg, a smartphone, tablet, or computer). Research assistants will directly invite providers at each of the participating pediatric or adolescent and adult clinics. Enrolled participants will complete a baseline survey and be oriented to *iTransition* and create a user account. During the 18-month intervention period, provider intervention participants will complete surveys at 6, 12, and 18 months, and a subset (up to 12) will be invited to participate in up to 3 qualitative interviews based on *iTransition* use (high and low/no). Overall, participants can receive up to US $100 to US $175 (US $25 per survey and qualitative interview) for their time over the course of the study.

#### Transition Champion Group

Inclusion criteria for the Transition Champion group include the same criteria as provider participants and nomination by a clinic staff member in their clinic to be an *iTransition* intervention point person (champion) who will support *iTransition* intervention use in their clinics. Following the baseline survey, orientation to *iTransition*, and a brief training, Transition Champions will complete surveys at 6, 12, and 18 months and be invited to participate in 3 qualitative interviews. Given the intensity and duration of their participation across the project, Transition Champions will receive a total of US $350 (US $50 for each study visit or interview) for their active support of *iTransition* use in their respective clinics by youth and provider groups.

### Sample Size and Power

The goals of this study are to estimate effect sizes so that we can fully power a future randomized control trial and to evaluate *iTransition* acceptability and feasibility. Our youth sample size is sufficient to detect large differences in our primary HCT outcome (linkage to adult care) between those using *iTransition* compared with the historical controls. Our sample size of 50 subjects per group provides 87% power to detect a 30% difference in HCT rates between the historical control and intervention groups, 71% power to detect a 25% difference, and 52% power to detect a 20% difference. These calculations are based on an estimated HCT rate in the historical control group of 40% (over the 12-month period), as our previous work showed that 37% of youth living with HIV transitioned into adult-oriented care settings within a 9-month period [6].

### Data Collection

Data collection for surveys and electronic medical record abstraction will occur in Research Electronic Data Capture [28]. Qualitative interviews will be digitally recorded, transcribed by a professional agency, and analyzed by the study team. Paradata (automatically collected electronic process data, eg, the number of times that a participant logged into the app) will be captured for all youth and provider intervention participants.

#### Youth Historical and Intervention Groups

Youth participants will complete a baseline survey with questions related to demographics, general health status, health behaviors (eg, substance use and medication adherence), Social Cognitive Theory–related factors, and other psychosocial covariates affecting HCT (eg, stigma, discrimination, social support, self-efficacy, skills, and readiness). The majority of measures have been validated and used in previous research (Multimedia Appendix 2 [29-44]). In line with the Social Cognitive Theory, we developed a measure to assess youth’s HCT-specific cues to action (eg, reminders to take medication and refill prescriptions, provider discussions related to HCT, provider-initiated skill development for adult clinic, and developing a written a HCT plan with the provider). We are also assessing incarceration history.

Intervention youth will also complete subsequent surveys at 6 and 12 months, including all the same baseline measures (except demographics). A subset of intervention youth will be invited to complete interviews related to *iTransition* use (eg, perceptions, experience, challenges, and recommendations for improving the intervention). Overall *iTransition* feasibility (ease of use), acceptability (tolerance of use), and use (eg, number of times in app and time with activities) data will be collected through survey and paradata.

We will review the electronic medical charts of youth in both study groups for a 3-year period: 12 months before baseline through 24 months after baseline. The use of medical chart data helps address potential attrition over time by allowing the assessment of outcomes (eg, medial appointment attendance and viral suppression). Chart reviews will be completed at 3 time points: baseline, 12 months after baseline, and 24 months after baseline. Each abstraction will capture data from the previous 12 months (ie, initial abstraction includes the 12 months before baseline, 12-month abstraction covers baseline to 12 months, and 24-month abstraction includes data between 12 months and 24 months). Information abstracted will include HIV history (eg, date of diagnosis, antiretroviral therapy information, and other sexually transmitted infection [STI] diagnoses), viral load, CD4+ count and percentage, diagnoses of STIs, and appointment information at pediatric or adolescent and adult clinics.

The primary clinical outcome variable for youth, measured at the patient level, is linkage to adult care (defined dichotomously as having 1 completed adult clinic appointment or not). Secondary clinical outcomes are care retention (dichotomously defined as having or not having 1 visit in each 6-month period) and viral suppression (<200 copies/mL) at 1 year after baseline. We will explore whether individual characteristics (eg, gender, race or ethnicity, and sexual orientation), Social Cognitive Theory–related covariates (eg, self-efficacy), and intervention dosage (as quantified by paradata metrics) are associated with outcomes or whether there is any preliminary evidence that it moderates intervention effects.

#### Provider and Transition Champion Groups

Data collection for the provider intervention and Transition Champion groups includes baseline and follow-up (at 6, 12, and 18 months) surveys (Multimedia Appendix 2). These will collect information related to demographics and professional experience (baseline only), clinic assessment (eg, leadership and self-efficacy to support HCT), and *iTransition* evaluation (Multimedia Appendix 2). Similar to the youth measures, the majority of provider measures are validated and have been used previously. The one measure that was not previously validated is related to general perceptions of the intervention (eg, barriers to intervention implementation, helpfulness of the intervention for supporting HCT, and motivations to use the intervention). A subset of providers and all of the Transition Champions will also be invited to complete interviews related to *iTransition* use (eg, perceptions, experiences, challenges, and recommendations for improving the intervention). Similar to youth intervention participants, overall *iTransition* use will be collected through survey and paradata. Primary outcomes for providers will be related to the use, feasibility, and acceptability of *iTransition*. In addition, changes in providers’ HCT-related self-efficacy will be examined.

### Outcome Analyses

#### Quantitative Analyses: Youth Data

To estimate intervention efficacy, we will use a logistic regression model with a term for intervention status to compare the likelihood of being linked to adult care between the intervention and historical control groups. We will conduct univariable and multivariable logistic regression analyses to test the intervention effects on our secondary outcomes of care retention and viral suppression after 12 months. Retention for patients who never achieve HCT or who undergo HCT too late in the 12-month follow-up period to calculate the retention outcome will be treated as missing. Other outcomes will include acceptability and feasibility of the app, app use metrics, and Social Cognitive Theory–related measures, such as self-efficacy for managing HCT.

#### Quantitative Analyses: Provider and Transition Champion Data

We will conduct descriptive statistics to assess the feasibility, acceptability, and use of *iTransition*. We will also conduct regression analyses to assess changes in providers’ self-efficacy in facilitating HCT for their patients over time.

#### Qualitative Analyses: Youth, Provider, and Transition Champion Data

Our interview data will help describe participants’ experiences with *iTransition* and understand the processes of implementing the intervention in different clinical environments. Qualitative analyses will be guided by content analysis, which is well suited for understanding participants’ experiences and comparing experiences within and across groups (eg, comparing youth from Atlanta and Philadelphia or comparing pediatric or adolescent and adult providers). We will develop a preliminary codebook [45] to include predetermined deductive codes related to the Social Cognitive Theory domains of interest and inductive codes that emerge from the data. Transcripts will be coded by 2 members of the research team, and differences will be discussed and resolved in team meetings until consensus is reached. Data analysis will also involve generating frequencies of codes and comparing the frequency of code occurrence across participant subgroups. To further establish the trustworthiness and validity of our data, we will present findings back to our youth design team members (who have recently undergone HCT and who will participate in the development and usability testing of *iTransition*).

## Results

Phase 1 (development) of *iTransition* research activities began in November 2019 and is ongoing. The data collection for the phase 2 pilot implementation trial is expected to be completed in January 2023. Final results are anticipated in summer 2023.

## Discussion

Although every youth in pediatric or adolescent HIV care will need to transition to adult-oriented care, there are no evidence-based HCT interventions [4,6]. HCT poses a persistent challenge to the health of youth living with HIV as they may disengage from care resulting in gaps in medication adherence and viral rebound [7]. Thus, interventions to support youth and providers at both pediatric or adolescent and adult clinics are crucial. *iTransition*, as a theory-based, stakeholder-engaged, multilevel mHealth intervention, is particularly poised to fill this important gap in HCT for youth and providers.

## Appendix

Multimedia Appendix 1 Screenshots of iTransition.

Multimedia Appendix 2 Primary and other outcome measures: operationalization and schedule.

## Abbreviations

CHOP: Children’s Hospital of Philadelphia
HCT: health care transition
IRB: Institutional Review Board
mHealth: mobile health
STI: sexually transmitted infection
