# Supplementary material for: An Intervention for the Transition From Pediatric or Adolescent to Adult-Oriented HIV Care: Protocol for the Development and Pilot Implementation of iTransition
Source: JMIR Res Protoc. 2021 Apr 7;10(4):e24565. doi: 10.2196/24565 (PMC8075294; doi:10.2196/24565)
Supplement: Multimedia Appendix 1 [file resprot_v10i4e24565_app1.docx]

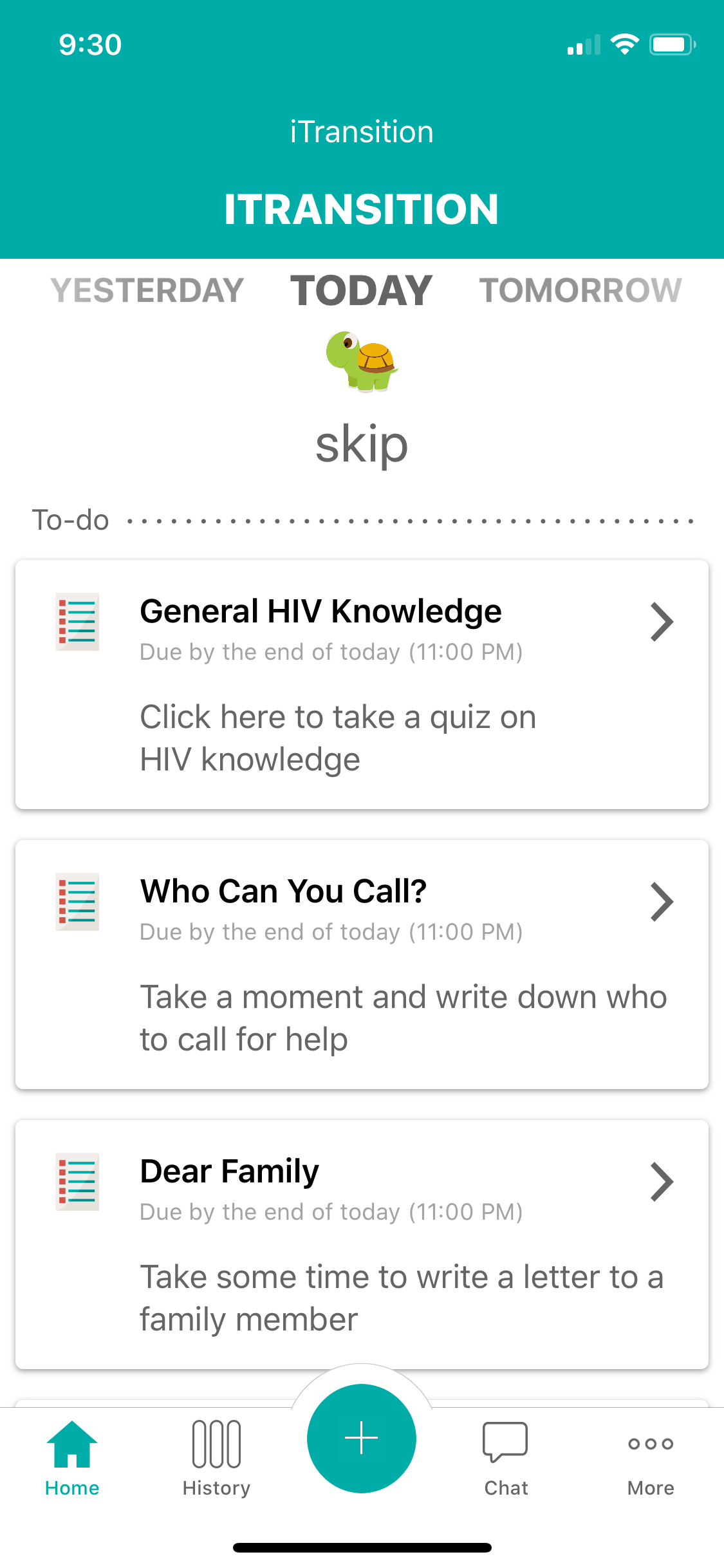

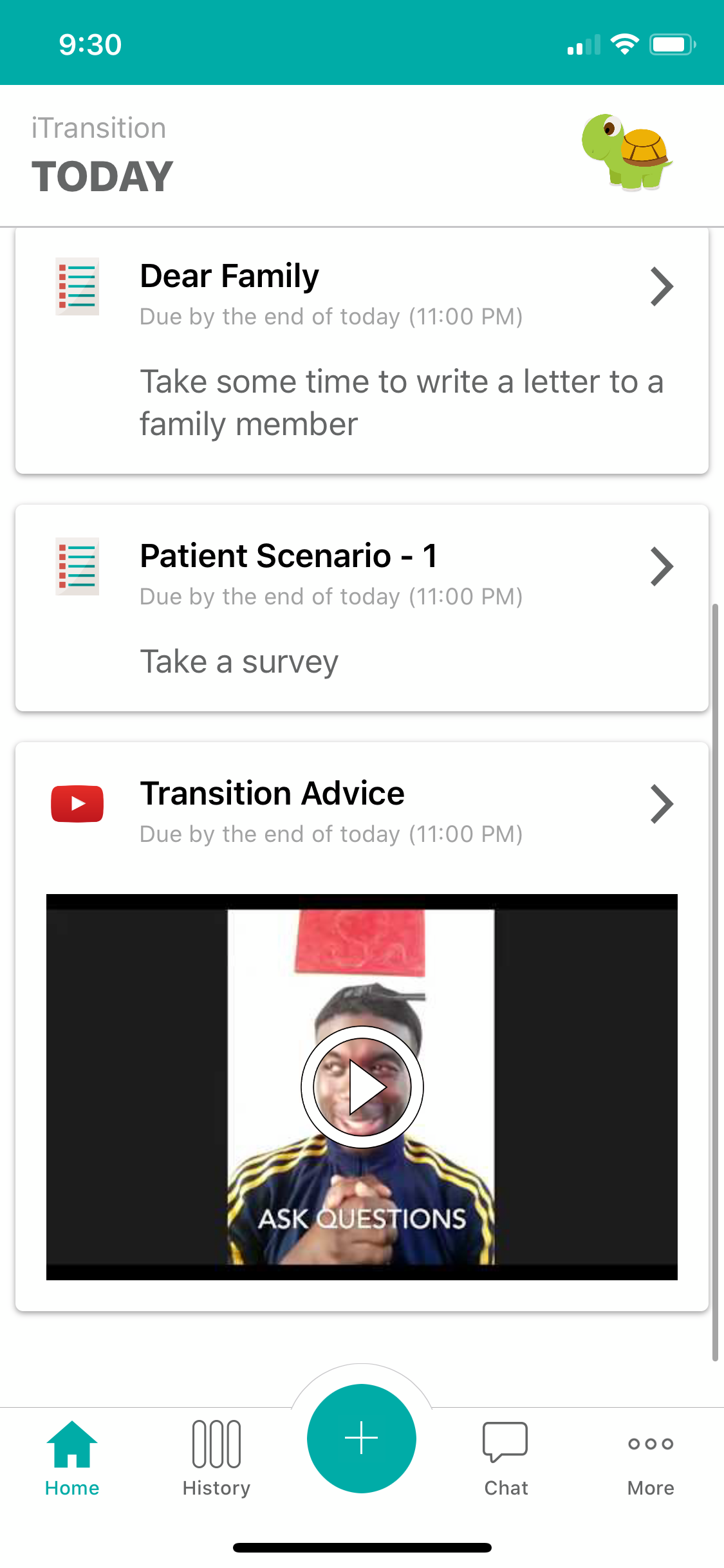

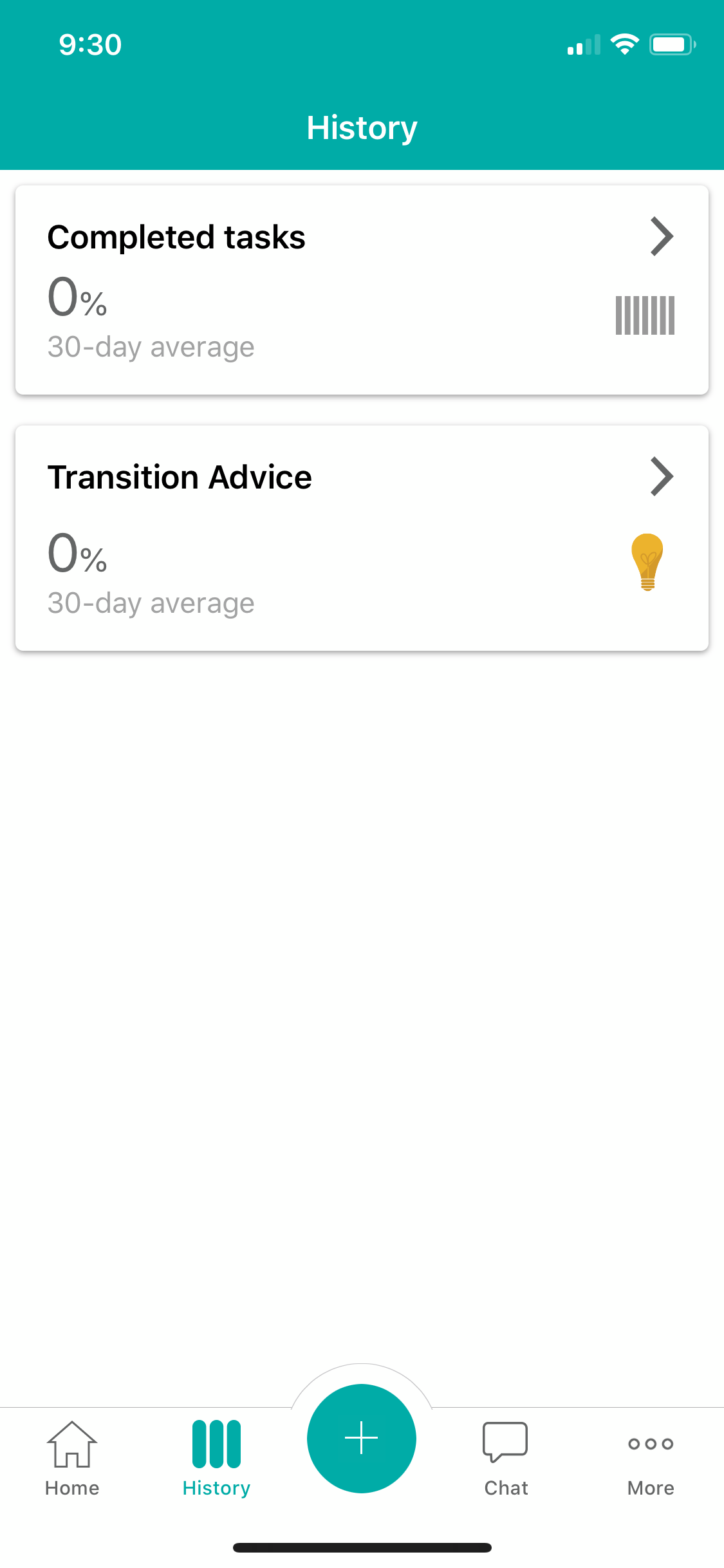


3. Image 3 shows the “history” screen of the app. It shows the record of completed tasks and the percentage of videos watched

2. Image 2 shows the “learn” screen of the app. It includes a To-Do list, which consists of activities for patients to complete and a video to watch

1. Image 1 shows the “home” screen of the app. It includes a To-Do list, which consists of activities for patients to complete


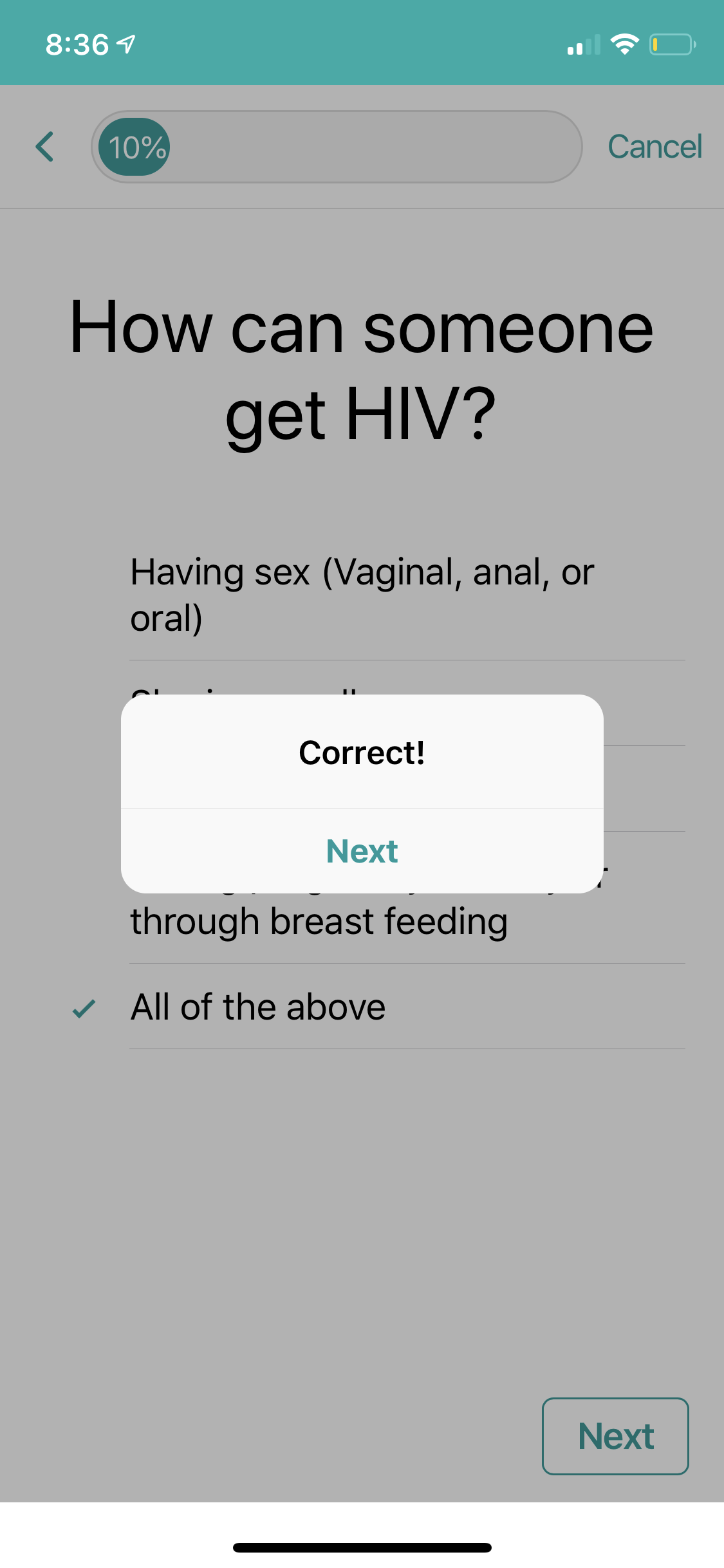

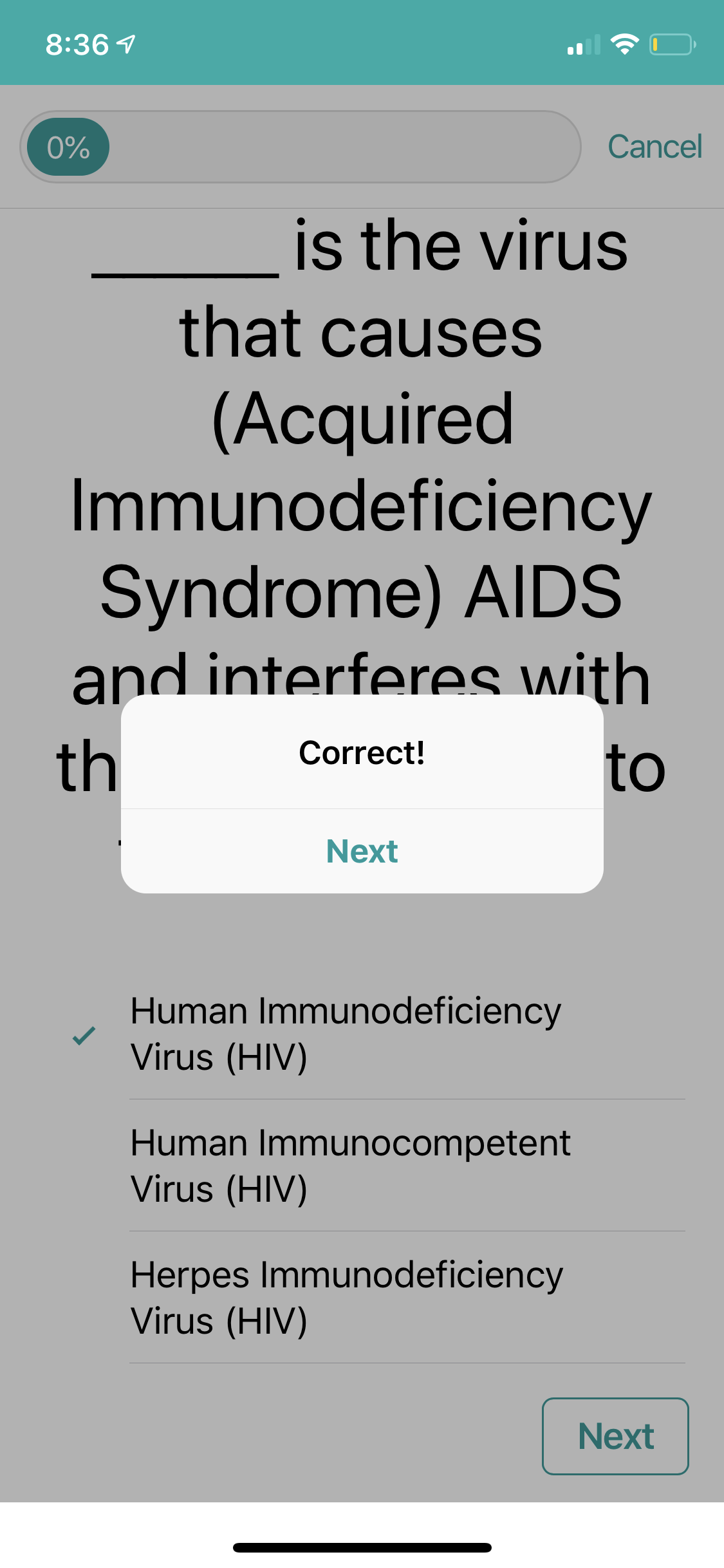

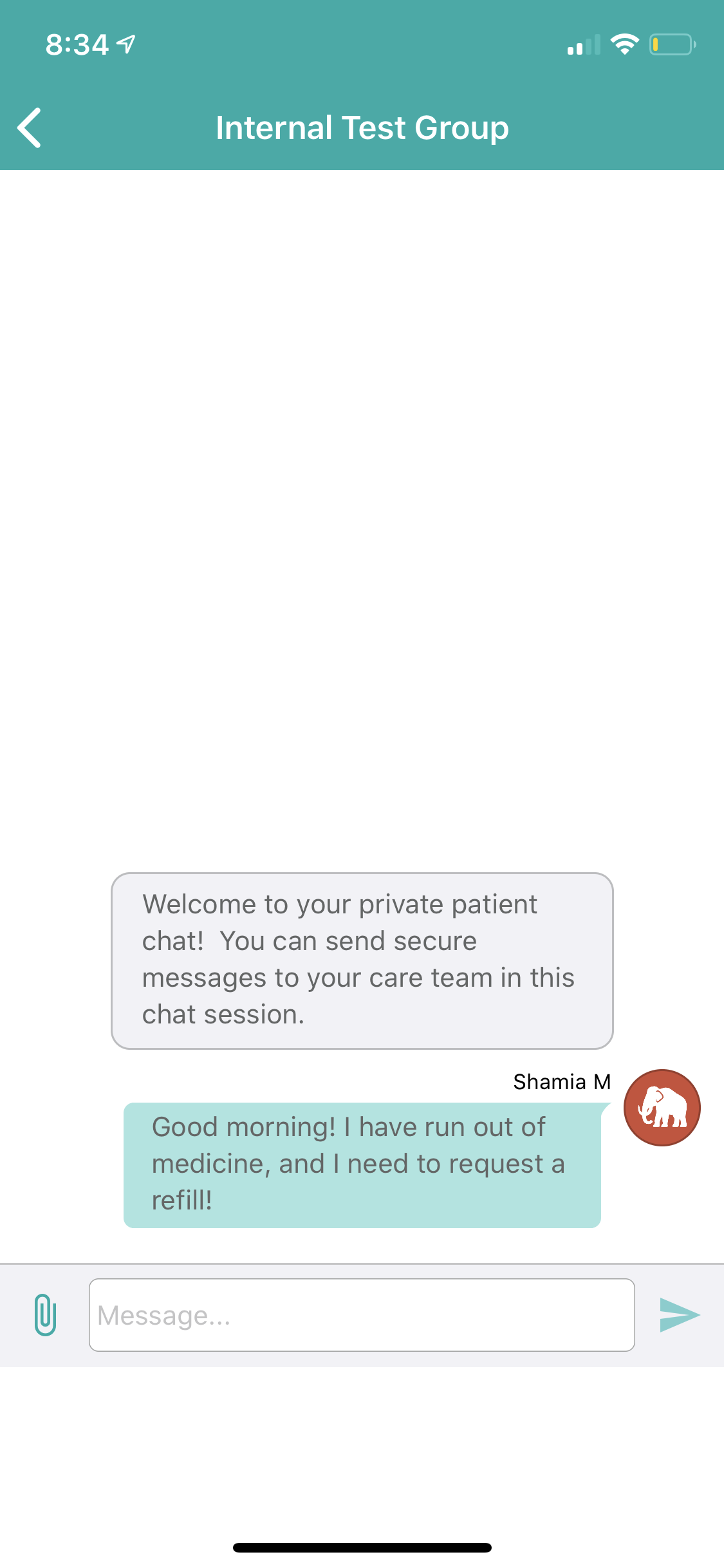


6. Image 6 shows the chat feature. The patient avatar (elephant) is shown

4. Image 4 shows an example of a correctly answered quiz question

5. Image 5 shows an example of a correctly answered quiz question


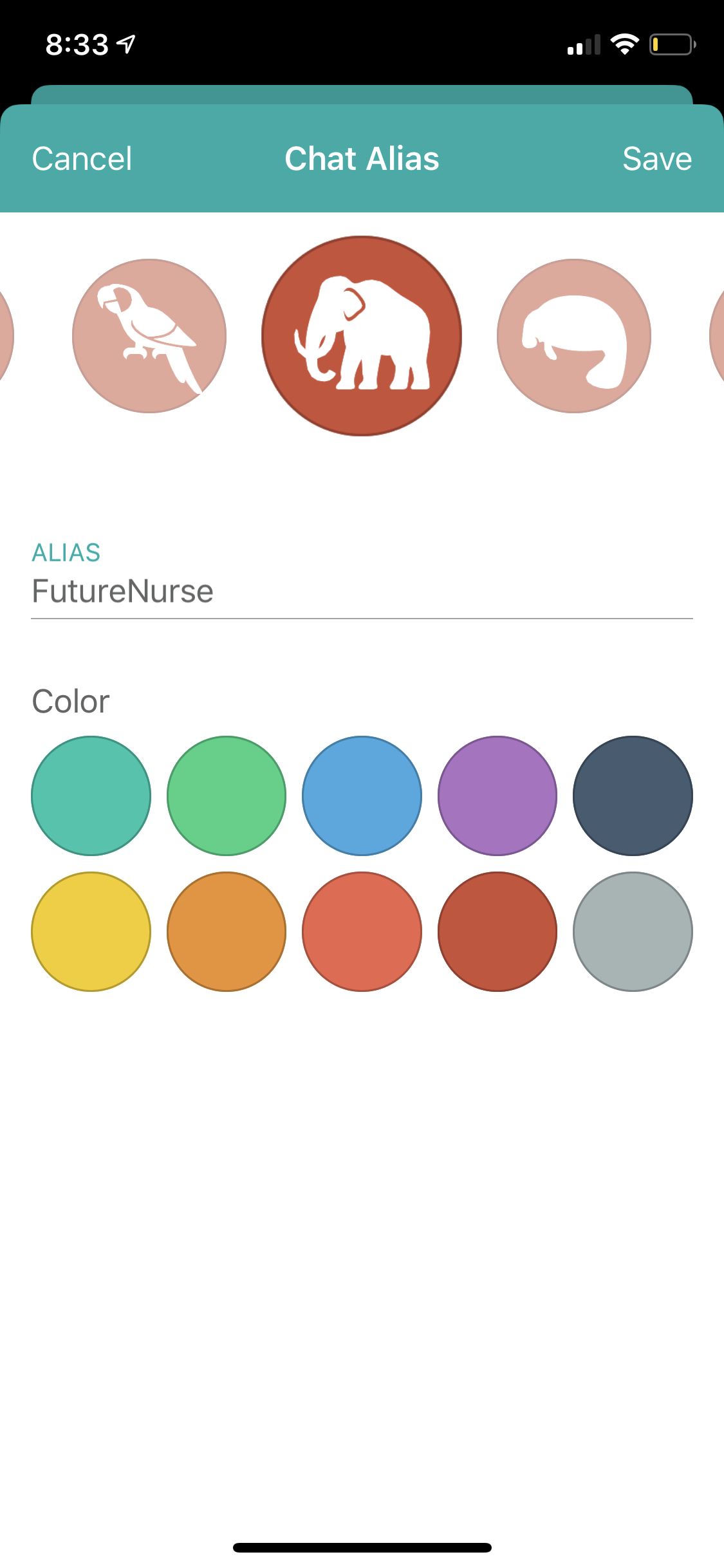

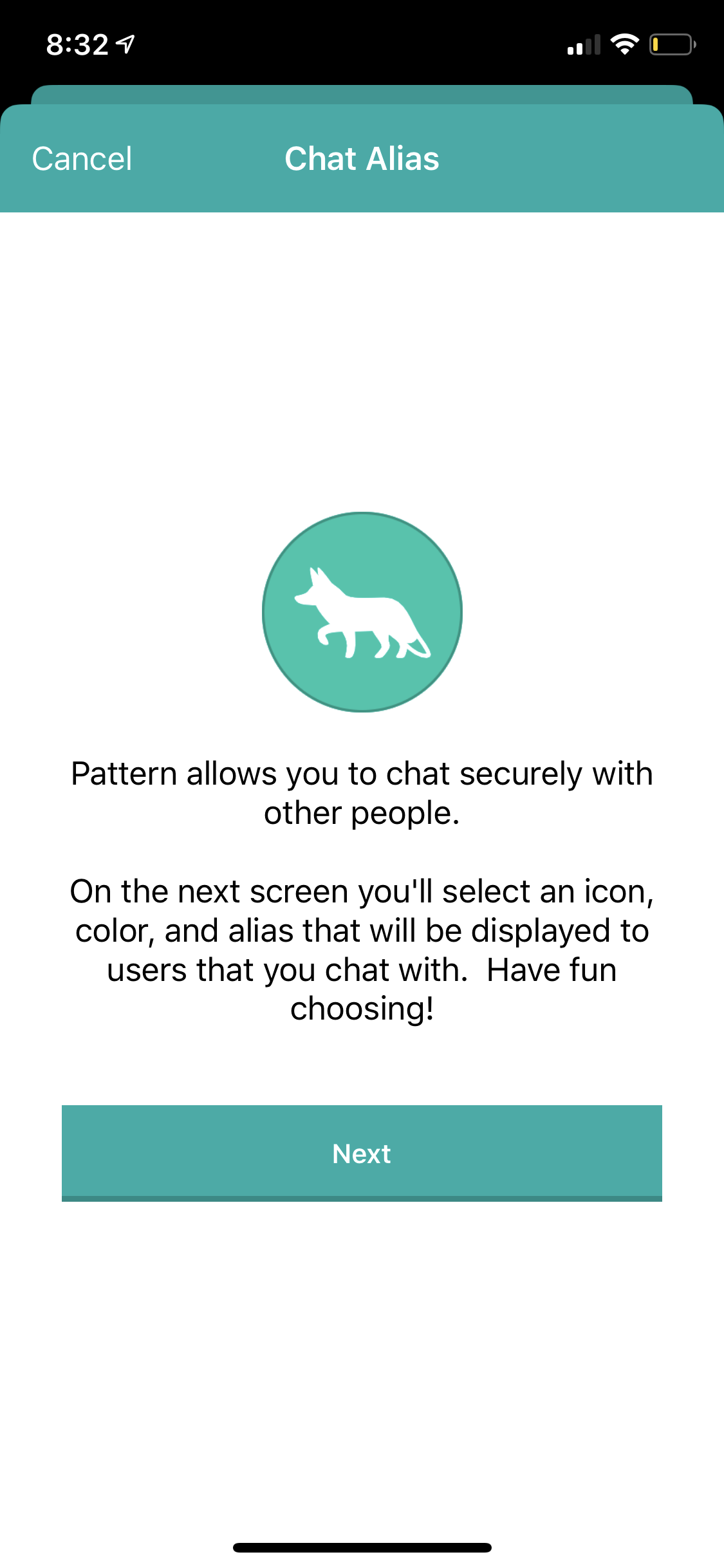

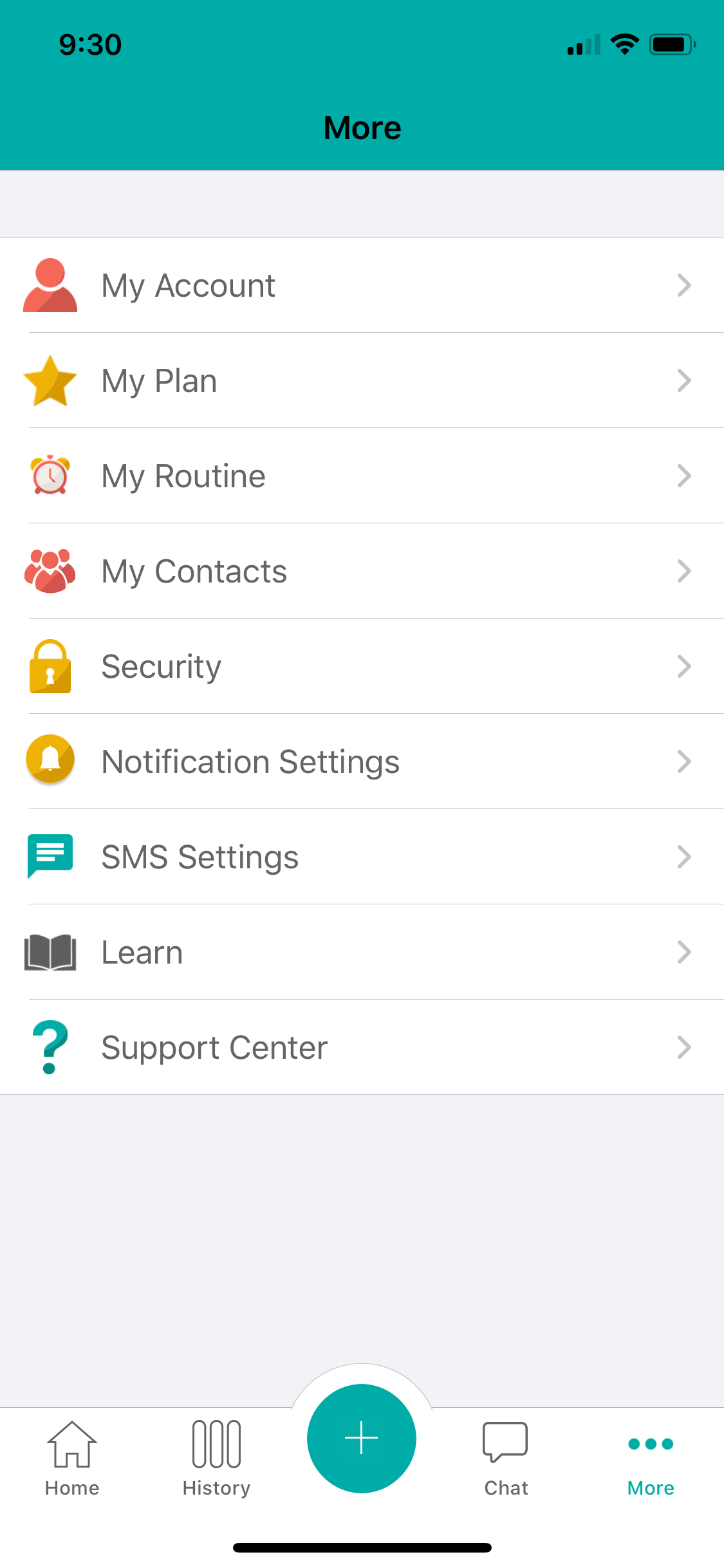


7. Image 7 shows the process of creating a chat alias

9. Image 9 shows the “more” screen, which includes several features

8. Image 8 shows what patients see when they first click the “chat” screen
