# Supplementary material for: An Intervention for the Transition From Pediatric or Adolescent to Adult-Oriented HIV Care: Protocol for the Development and Pilot Implementation of iTransition
Source: JMIR Res Protoc. 2021 Apr 7;10(4):e24565. doi: 10.2196/24565 (PMC8075294; doi:10.2196/24565)
Supplement: Multimedia Appendix 2 [file resprot_v10i4e24565_app2.docx]

**Multimedia Appendix 2.** Primary and other outcomes measures: operationalization and schedule.

| Participants | | | Measures and operationalization | Measurement schedules | | | | |
| --- | --- | --- | --- | --- | --- | --- | --- | --- |
|  | | |  | Baseline^a^ | 6 months | 12 months | 18 months | 24 months |
|  | | |  |  |  |  |  |  |
| **Youth participants** | | | | | | | | |
|  | **Health behaviors** | | | | | | | |
|  |  | HIV medication adherence | Medication adherence in the past 30 days (VAS^b^) [29] and the past 4 days (ACTG^c^) [30] | X^d^ | X | X | —^e^ | — |
|  |  | Recreational substance use | Substance use in past 3 months and lifetime [31] | X | X | X | — | — |
|  |  | Incarceration history | Incarceration in past 3 months and lifetime | X | X | X | — | — |
|  | **Health: overall, psychological, and developmental** | | | | | | | |
|  |  | HIV health perceptions | Quality of life and health (eg, perceptions, health distress, and social and cognitive functioning) for individuals living with HIV: MOS^f^-HIV-35 items [32] | X | X | X | — | — |
|  |  | Stigma | Experiences because of the stigma associated with HIV-positive status: Brief Stigma Scale-10 items [33] | X | X | X | — | — |
|  |  | Mental health | Assessment of anxiety, depression, and somatization symptoms and severity: Brief Symptoms Inventory-18 items [34] | X | X | X | — | — |
|  |  | Social support | General social support: MOS Social Support-19 items [35] | X | X | X | — | — |
|  |  | Discrimination | Chronic minor experiences of unfair treatment: Everyday Discrimination Scale-5 items [36] | X | X | X | — | — |
|  |  | Electronic health information | Knowledge, skills, and comfort with electronic health information: eHEALS^g^-8 items [37] | X | X | X | — | — |
|  | **Social Cognitive Theory constructs** | | | | | | | |
|  |  | Self-management skills and self-efficacy: health care transition | Self-management skills and self-efficacy for HIV care: TRAQ^h^-30 items [38] | X | X | X | — | — |
|  |  | Outcome expectancies | Treatment expected to receive at an adult clinic: SETS^i^-5 items [39] | X | X | X | — | — |
|  |  | Self-efficacy: HIV treatment adherence | Confidence in ability to carry out HIV treatment plan (eg, medication regimen adherence and nutrition): HIV-ASES^j^-12 items [40] | X | X | X | — | — |
|  |  | Cues to action | Cues received to help with HIV-related care management: yes/no questions | X | X | X | — | — |
|  | **Intervention evaluation** | | | | | | | |
|  |  | *iTransition* web app evaluation | Acceptability and quality of *iTransition*: uMARS^k^-20 items [41] | — | X | X | — | — |
|  | **Medical record information** | | | | | | | |
|  |  | HIV history | Date of HIV diagnosis, first HIV medical appointment, and ART^l^ prescription date of initiation | X | — | X^a^ | — | X^a^ |
|  |  | HIV outcomes and STI^m^ diagnosis | HIV laboratories for viral load and CD4 count/percent; STI laboratory test for chlamydia, gonorrhea, and syphilis | X | — | X^a^ | — | X^a^ |
|  |  | HCT^n^ care linkage and engagement | Scheduled appointments attended and missed | X | — | X^a^ | — | X^a^ |
| **Provider and Transition Champion participants** | | | | | | | | |
|  | **Clinic assessment** | | | | | | | |
|  |  | Self-efficacy: health care transition | Self-reported self-efficacy for youth skill development, clinic environment, and inter-clinic staff relationship: HHCTSE^o^-16 items | X | X | X | X | — |
|  |  | Transition readiness | Motivation and capacity of integrating *iTransition* at a tool for HCT: RICQ^p^-15 items [42] | X | X | X | X | — |
|  |  | Leadership | Knowledge, attitudes, and behaviors related to HCT best evidence-based practices: ILS^q^-12 items [43] | X | X | X | X | — |
|  | **Intervention evaluation** | | | | | | | |
|  |  | Perception of *iTransition* | General perceptions of the intervention: 36 items | X | X | X | X | — |
|  |  | *iTransition* web app evaluation | Acceptability and quality of *iTransition*: uMARS-20 items [41] | — | X | X | X | — |
|  |  | Acceptability and feasibility | Acceptability, appropriateness, and feasibility: AIM^r^, IAM^s^, and FIM^t^-12 items [44] | — | X | X | X | — |

^a^Youth historical control group completes all the baseline measures and will have medical record information abstracted at all the time points.

^b^VAS: Visual Analogue Rating Scale

^c^ACTG: AIDS Clinical Trials Group

^d^Survey included in scheduled assessment

^e^Survey not included in scheduled assessment

^f^MOS: Medical Outcomes Study

^g^eHEALS: eHealth Literacy Scale

^h^TRAQ: Transition Readiness Assessment Questionnaire

^i^SETS: Stanford Expectations of Treatment Scale

^j^ASES: Adherence Self-Efficacy Scale

^k^uMARS: User Version of the Mobile Application Rating Scale

^l^ART: antiretroviral therapy

^m^STI: sexually transmitted infection.

^n^HCT: health care transition.

^o^HHCTSE: HIV Health Care Transition Provider Self Efficacy Scale

^p^RICQ: Readiness for Integrated Care Questionnaire

^q^ILS: Implementation Leadership Scale

^r^AIM:Acceptability of Intervention Measure

^s^IAM: Intervention Appropriateness Measure

^t^FIM: Feasibility of Intervention Measure
